# Nicotinamide N‐methyltransferase is related to MELF pattern invasion in endometrioid carcinoma

**DOI:** 10.1002/cam4.4359

**Published:** 2021-10-16

**Authors:** Shinichiro Tahara, Satoshi Nojima, Kenji Ohshima, Yumiko Hori, Kazuaki Sato, Masako Kurashige, Takahiro Matsui, Daisuke Okuzaki, Eiichi Morii

**Affiliations:** ^1^ Department of Pathology Osaka University Graduate School of Medicine Osaka Japan; ^2^ Single Cell Genomics Human Immunology WPI Immunology Frontier Research Center Osaka University Osaka Japan; ^3^ Genome Information Research Center Research Institute for Microbial Diseases Osaka University Osaka Japan; ^4^ Institute for Open and Transdisciplinary Research Initiatives Osaka University Osaka Japan

**Keywords:** endometrioid carcinoma, invasion, MELF, migration, NNMT

## Abstract

Grade 1 (G1) endometrioid carcinoma (EC) is relatively a good prognosis. However, in a minority of cases, G1 shows an aggressive histological pattern known as the microcystic, elongated, and fragmented (MELF) pattern. We previously reported that EC with high expression levels of S100A4 and serum deprivation‐response protein (SDPR) was related to MELF pattern invasion. However, the molecular features of the invasive front area of the MELF pattern have not been investigated. In this study, we searched for genes preferentially expressed in the invasive front area of EC with the MELF pattern using laser microdissection and RNA sequencing, and showed that nicotinamide N‐methyltransferase (NNMT) is related to MELF pattern invasiveness. Immunohistochemical analyses confirmed high NNMT expression in the invasive front area of the MELF pattern. Moreover, NNMT promoted migration, invasion, colony formation, epithelial–mesenchymal transition (EMT), and chemoresistance using EC cell lines. We speculate that depletion of NNMT promotes histone methylation and leads to tumor suppression because NNMT consumes S‐adenosyl methionine (SAM), which is an essential methylation cofactor. NNMT knockout cells showed enhanced expression of H3K9me2. RNA sequencing using NNMT knockout cell lines suggested that methylation of H3K9 leads to repression of the transcription of various oncogenic genes. Our findings demonstrate the possibility that NNMT inhibitors, which are expected to be used for the treatment of metabolic disorders, would be effective for the treatment of aggressive EC. This is the first report of gene analyses focusing on the morphological changes associated with MELF pattern invasion of EC.

## Graphical abstract

We searched for genes preferentially expressed in the invasive front area of EC with the MELF pattern using laser microdissection and RNA sequencing. We showed that NNMT is related to MELF pattern invasiveness.

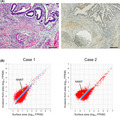

## 1 INTRODUCTION

Endometrioid carcinoma (EC) is one of the most common histological types of uterine corpus cancers. The International Federation of Gynecology and Obstetrics (FIGO) classifies EC into three groups, that is, grade 1 (G1), grade 2 (G2), and grade 3 (G3), in increasing order of the proportion of solid components. In general, G1 is associated with a good prognosis. However, in a minority of cases, G1 shows an aggressive histological pattern known as the microcystic, elongated, and fragmented (MELF) pattern. The MELF pattern was first described by Murray et al. and was reported to be associated with a fibromyxoid reaction, vascular invasion, and lymph node metastasis.^1^ Stewart et al. showed that the MELF pattern had features of the epithelial–mesenchymal transition (EMT) in immunohistochemical analyses.^2^ Moreover, we reported that EC with a high level of S100A4 and serum deprivation‐response protein (SDPR) expression was related to MELF pattern invasion.^3^, ^4^ However, the molecular features of the invasive front area of the MELF pattern have not been investigated.

The present study searched for genes preferentially expressed in the invasive front area of EC with the MELF pattern using laser microdissection and RNA sequencing. Nicotinamide N‐methyltransferase (NNMT) is the enzyme responsible for catalyzing the methylation of nicotinamide using the methyl donor S‐adenosyl methionine (SAM) to produce S‐adenosyl‐L‐homocysteine (SAH) and 1‐methylnicotinamide (1‐MNA). Physiologically, hepatocytes express the highest levels of NNMT.^5^ On the other hand, NNMT expression is increased in a wide variety of cancers, such as colorectal cancer, gastric cancer, hepatocellular carcinoma, lung cancer, and oral squamous cell carcinoma.^6^, ^7^ Hence, we also examined the function of NNMT using EC cell lines.

## 2 MATERIALS AND METHODS

### 2.1 Patients

We examined 120 surgical cases for EC of the uterine corpus at Osaka University Hospital between 2011 and 2019. The histological subtypes were G1 with MELF pattern invasion, G1 without MELF pattern invasion, G2, and G3 (*n* = 30, respectively). This study was approved by the Ethics Review Board of the Graduate School of Medicine, Osaka University (No. 15234).

### 2.2 RNA sequencing analyses of FFPE samples and ingenuity pathway analysis

**FIGURE 1:**
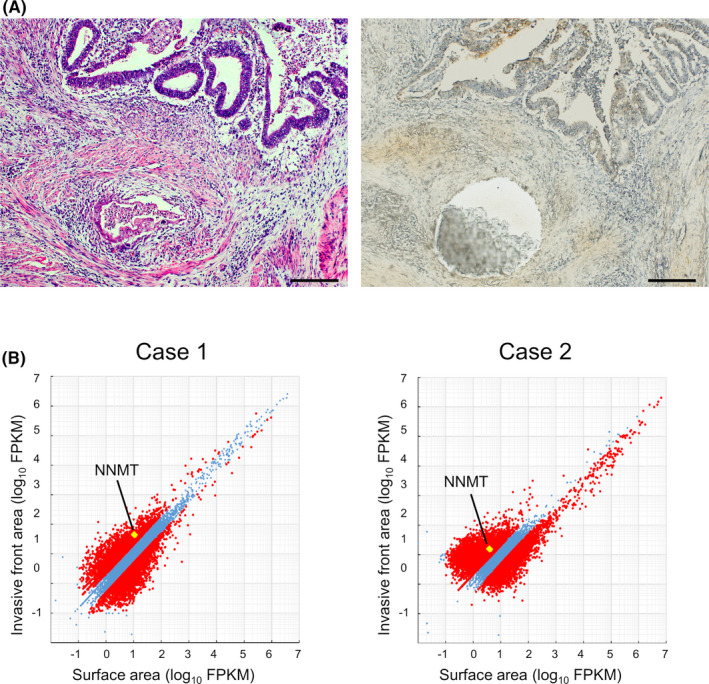
RNA sequencing analyses of FFPE samples using laser microdissection. (A) Representative hematoxylin and eosin (H&E) staining of EC with MELF pattern invasion (left). Representative images of laser microdissection for isolation of the invasive front area (right). Scale bar: 200 μm. (B) RNA sequencing results plotted as a scatterplot matrix. NNMT was expressed higher in the invasive front area than in the surface area in both cases

From 30 cases of EC with MELF pattern invasion, we selected 2 cases, in which they were morphologically typical and had a sufficient invasive front area, and submitted them for RNA sequencing analyses. To compare the gene expression profiles between the invasive front area and the surface area, laser microdissection was performed (Leica LMD 7000; Leica Microsystems, Wetzlar, Germany) (Figure 1A). Formalin‐fixed paraffin‐embedded (FFPE) sections (10 µm) thick were cut and mounted on laser microdissection slides (DIRECTOR; AMR, Tokyo, Japan). Tumor cells in the invasive front area and the surface area were microdissected separately in each case and subjected to RNA sequencing analyses. Total RNA was extracted from the cells using an miRNeasy FFPE Kit (Qiagen, Hilden, Germany) in accordance with the manufacturer's protocol. NGS library preparation was performed using the SMARTer® Stranded Total RNA Sample Prep Kit‐Pico Input Mammalian (Clontech, TaKaRa) according to the manufacturer's instructions. Sequencing was performed on an Illumina HiSeq 2500 platform in 75‐base single‐end mode with Illumina Casava 1.8.2 software for base calling (Illumina, San Diego, CA). Sequenced reads were mapped to the human reference genome sequence (hg19) using TopHat version 2.0.13, in combination with Bowtie 2 version 2.2.3 and SAMtools version 0.1.19. Fragments per kilobase of exon per million mapped fragments (FPKM) were calculated using Cufflinks version 2.2.1. Raw data have been deposited in the NCBI Gene Expression Omnibus database (GSE171033).

The genes identified by RNA sequencing analyses were subjected to ingenuity pathway analysis (IPA) (Qiagen) to identify pathways related to the invasive front area of the MELF pattern. We identified 110 genes showing significantly higher expression in the invasive front area (fold change >5 and FPKM of invasive front area >10 in both cases) and 96 genes with significantly lower expression in the invasive front area (fold change <–5 and FPKM of surface area >10 in both cases) (Tables S1 and S2). A total of 206 genes were subjected to core analyses, and we targeted the canonical pathway.

### 2.3 Immunohistochemistry

Immunostaining was performed using a Ventana BenchMark GX Autostainer (Roche, Basel, Switzerland). Staining intensity (0, 1+, 2+, or 3+) was determined for each sample independently by two pathologists (ST and EM). Histological score (H‐score) was calculated using the following formula: [1 × (% tumor cells of 1+) +2 × (% tumor cells of 2+) +3 × (% tumor cells of 3+)].

### 2.4 Cell lines

The human EC cell lines AN3CA, HEC1A, HEC1B, HEC108, HEC116, and SNG‐M were obtained from Health Science Research Resources Bank (Osaka, Japan). Cells were cultured in DMEM with 10% FBS.

### 2.5 Generation of NNMT knockout AN3CA cells using the CRISPR/Cas9 system

Seventy percent confluent AN3CA cells were co‐transfected with equal amounts of NNMT CRISPR/Cas9 knockout plasmid and NNMT HDR plasmid (sc‐403192 and sc‐403192‐HDR, respectively; Santa Cruz Biotechnology) using Lipofectamine 3000 reagent (Thermo Fisher Scientific) and incubated for 72 h. Transfected cells were suspended in PBS supplemented with FBS (2%). Green and red populations were sorted into 96‐well plates in single‐cell sorting mode with SH800ZDP (Sony Imaging Products & Solutions Inc.) cell sorter. We constructed stable NNMT knockout (KO1 and KO2) cells. Similarly, we co‐transfected control CRISPR/Cas9 plasmid (sc‐418922; Santa Cruz Biotechnology) and NNMT HDR plasmid into AN3CA cells and selected stably transfected cells using the cell sorter in order to generate control cells (empty vector, EV).

### 2.6 Generation of HEC1B and HEC108 cells expressing GFP or NNMT‐GFP

The plasmids Empty‐GFP and NNMT‐GFP purchased by Vector Builder, Inc. were transfected into HEC1B and HEC108 cells using Lipofectamine 3000. Cells were subjected to proliferation, migration, and invasion assays 48 h after transfection. They were also subjected to colony formation assay and immunoblotting analyses 24 and 96 h after transfection, respectively.

### 2.7 Antibodies

An antibody against NNMT (sc‐376048; Santa Cruz Biotechnology) was used for immunoblotting (1:100) and immunohistochemistry (1:100). Antibodies for immunoblotting against ERK1/2 (1:1000; no. 4695), phospho‐ERK1/2 (Thr202/Tyr204) (1:2000; no. 4370), H3K4me3 (1:1000; no. 9751), and H3K27me3 (1:1000; no. 9733) were purchased from Cell Signaling Technology. Antibodies against neural cadherin (N‐cadherin) (1:200; sc‐59987; Santa Cruz Biotechnology) and H3K9me2 (1:500; ab1220; Abcam) were used for immunoblotting. An antibody against Cleaved Caspase‐3 (no. 9661; Cell Signaling Technology) was used for immunohistochemical analyses (1:400). Antibodies against β‐actin (1:1000; no. 5125) and Histone H3 (1:1,000; no. 4499), used as loading controls for immunoblotting, were obtained from Cell Signaling Technology.

### 2.8 Immunoblotting

Cells were lysed with sonication in buffer containing 10 mM 4‐(2‐hydroxyethyl)‐1‐piperazineethanesulfonic acid, 10 mM KCl, 1 mM ethylenediaminetetraacetic acid, 1 mM dithiothreitol, and 0.1% Nonidet P‐40. Electrophoresis was performed in 5%–20% gradient sodium dodecyl sulfate–polyacrylamide gels (ATTO), and proteins were transferred to polyvinylidene fluoride membranes (Merck). Primary antibodies were detected using a horseradish peroxidase‐conjugated anti‐mouse or anti‐rabbit IgG (H+L chain) (1:5,000; MBL). Images were obtained with an ImageQuant LAS 4000 (GE Healthcare). The results were quantified using ImageJ (https://imagej.nih.gov/ij/).

### 2.9 Cell block construction

For cell block construction, cells were centrifuged and washed with PBS. Neutral buffered formalin was gently applied. After 10 min, pellets were washed with PBS and washed three times with ethanol. Pellets were aspirated, collected in mesh biopsy bags, and routinely processed for paraffin inclusion. Immunohistochemical staining of cell blocks was performed using an autostainer.

### 2.10 Proliferation assay

To evaluate proliferation, cells were seeded at 1 × 10^5^ per well in 6‐well culture plates. After incubation for some days, cells were counted using the Muse Cell Analyzer (Merck).

### 2.11 Matrigel invasion assay

Tumor cell invasion was examined using the Corning BioCoat Matrigel Invasion Chamber (Corning Inc.). Tumor cells were placed in the upper chamber in DMEM without FBS and incubated at 37°C for 24 h. The lower chamber contained DMEM with 10% FBS. Invasive cells were stained with Diff‐Quik (Sysmex). The number of invasive cells was counted in five random fields per chamber at high magnification.

### 2.12 Wound healing assay

Confluent cells in 6‐well culture plates were wounded using CELL Scratcher (AGC TECHNO GLASS) and incubated in culture medium. The migration distance was calculated by subtracting the width of the wound at 40 or 50 h from that at 0 h.

### 2.13 Colony formation assay

Cells were applied to a pluriStrainer (pluriSelect). The collected cells were seeded at 4 × 10^3^ per well in 96‐well culture plates and cultured for 7 days in Cancer Stem Cell Media Premium (ProMab Biotechnologies).

### 2.14 Gelatin zymography

Cells were seeded and cultured in DMEM without 10% FBS for 12 h. Then, the supernatant was collected, concentrated using Vivaspin (GE, Fairfield), and subjected to gelatin zymography using a Gelatin Zymography Kit (Cosmo Bio). To verify equal protein concentration, the supernatant was analyzed using a BCA Protein Assay Kit (Takara Bio) and total protein was adjusted. The images were obtained with a ChemiDoc XRS Plus (Bio‐Rad Laboratories). The results were quantified using ImageJ.

### 2.15 Cell shape analyses

Cells were imaged using the BZ‐8000 microscope (Keyence). We selected 30 cells randomly, and the circularity of them was assessed using ImageJ. A circularity value of 1.0 indicates a perfect circle, whereas those approaching 0.0 indicate an increasingly elongated polygon.

### 2.16 Effects of anticancer drugs

Aliquots of 1 × 10^4^ cells in 96‐well plates were incubated for 24 h. We subsequently added cisplatin (Merck) (0, 1, 2, 4, 8, 16, 32, or 64 μM) or carboplatin (Merck) (0, 15.6, 31.3, 62.5, 125, 250, 500, or 1000 μM). After 48 h (cisplatin) or 72 h (carboplatin) of incubation, we added WST‐1 reagent (Takara Bio) and measured the absorbance using an SH‐9000 laboratory microplate reader (Corona Electric).

### 2.17 Cellular oxidative stress

CellROX Deep Red (Thermo Fisher Scientific) (5 μM) was added to the culture medium and incubated for 30 min. Cells were washed and suspended in PBS. They were analyzed by flow cytometry (FACSCanto II; BD Biosciences). Next, they were gated for singlets and the mean of the fluorescence was evaluated by FlowJo software. For cisplatin treatment, cells were incubated containing 8 μM cisplatin for 24 h. Then, CellROX Deep Red was added and assessed.

### 2.18 RNA sequencing analyses of AN3CA cells and ingenuity pathway analysis

To compare the gene expression profiles between NNMT knockout AN3CA cells and control cells, total RNA was extracted from KO1, KO2, and EV cells. Library preparation was performed using a TruSeq stranded mRNA sample prep kit (Illumina) according to the manufacturer's instructions. The subsequent procedures were as described above. Raw data have been deposited in the NCBI Gene Expression Omnibus database (GSE171032).

The genes identified by RNA sequencing were subjected to IPA (Qiagen) to identify pathways related to NNMT. We identified 345 genes with significantly higher expression in EV than in KO1 (fold change < –2 and FPKM of EV >2) and 289 genes with significantly higher expression in EV than in KO2 (fold change < –2 and FPKM of EV >2) (Tables S3 and S4). We separately subjected the results to core analyses. Comparative analyses were performed using the two datasets. We paid attention to Diseases and Bio Functions.

### 2.19 Statistical analyses

We performed statistical analyses using JMP Pro 14 software (SAS Institute). The results are presented as means ± SEs. The results were compared using the Student's *t‐*test, the Wilcoxon signed‐rank test, and analysis of variance (ANOVA), followed by the Dunnett's test. In all analyses, *p* < 0.05 was taken to indicate statistical significance.

## 3 RESULTS

### 3.1 NNMT expression level is high in the invasive front area of the MELF

**FIGURE 2:**
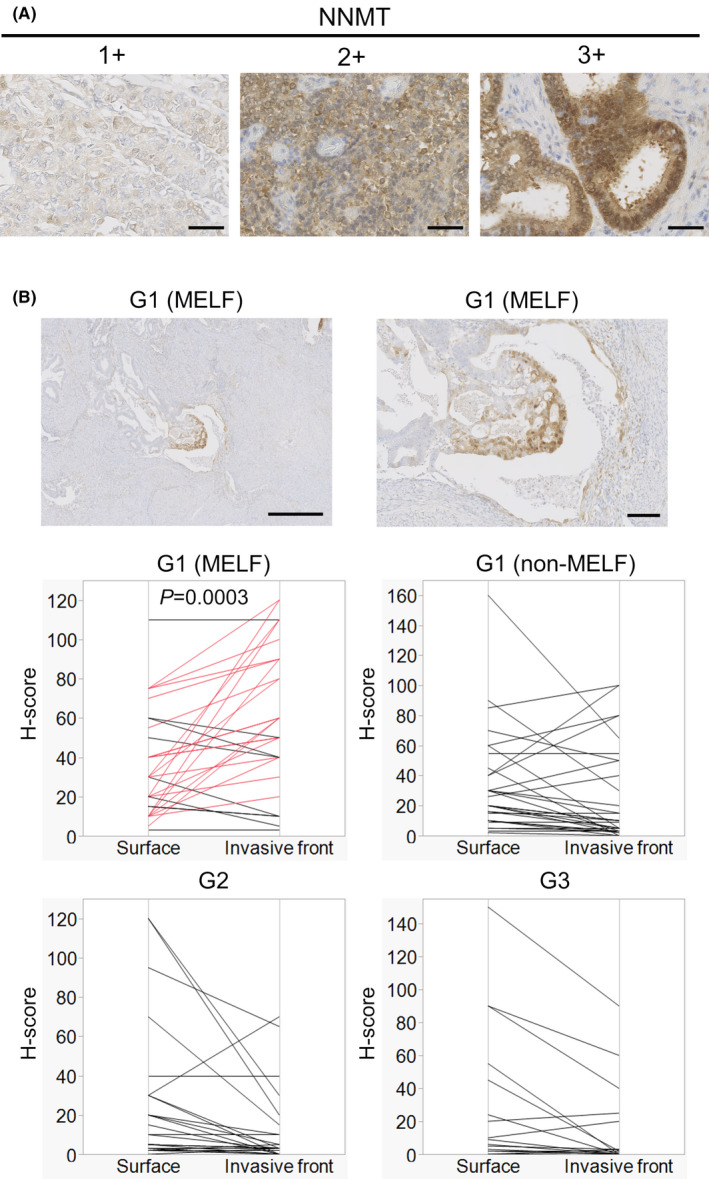
Immunohistochemistry of NNMT in EC with the MELF pattern. (A) Representative images of various staining intensity: 1+ (weak), 2+ (moderate), and 3+ (strong). (B) Representative images of immunohistochemical analyses of EC with the MELF pattern in the invasive front area (upper pictures). Comparison of H‐scores of the invasive front area and the surface area in G1 with MELF pattern invasion, G1 without MELF pattern invasion (non‐MELF), G2, and G3 (*n* = 30, respectively) (lower 4 graphs). Each line shows the H‐score of the invasive front area and the surface area in each case. In G1 with MELF pattern invasion, the data show an increase in the invasive front area in 21 of 30 cases (red lines). Scale bars: 50 μm in (A), 500 μm in the left upper picture of (B), and 100 μm in the right upper picture of (B). We used the Wilcoxon signed‐rank test to calculate *p*‐values

To elucidate the mechanism of the invasiveness of the MELF pattern, we performed RNA sequencing analyses of both the invasive front area and the surface area of MELF cases. IPA revealed that in the canonical pathways, the sirtuin signaling pathway was enhanced in the invasive front area (Table 1). The sirtuin signaling is known to be regulated by NNMT. Hong et al. reported that NNMT expression stabilizes sirtuin 1 protein and related signals in hepatic nutrient metabolism.^8^ The regulation is shown not only in normal organs, but also in tumor tissues, such as breast cancer, prostate cancer, neuroblastoma, and so on.^6^, ^9^, ^10^ Therefore, we focused on NNMT. NNMT expression was higher in the invasive front area than in the surface area (Figure 1B). To confirm this observation, we performed immunohistochemical analyses of tissue sections from EC with the MELF pattern. We show typical cases in Figure 2A. We compared NNMT expression between the invasive front area and the surface area in an individual case. The H‐score of the invasive front area was significantly higher than that of the surface area only in G1 with MELF cases (Figure 2B). This tendency was not found in G1 without MELF, G2, and G3. Thus, these observations suggested that high expression of NNMT relates to the invasiveness with the MELF pattern.

**TABLE 1:** List of top five canonical pathways analyzed by IPA

Name of canonical pathway	*p*‐value
Sirtuin signaling pathway	1.54E−04
GNRH signaling	5.73E−04
IL‐10 signaling	2.49E−03
Glucose and Glucose−1‐phosphate degradation	3.50E−03
Toll‐like receptor signaling	3.53E−03

### 3.2 NNMT expression detected in AN3CA

**FIGURE 3:**
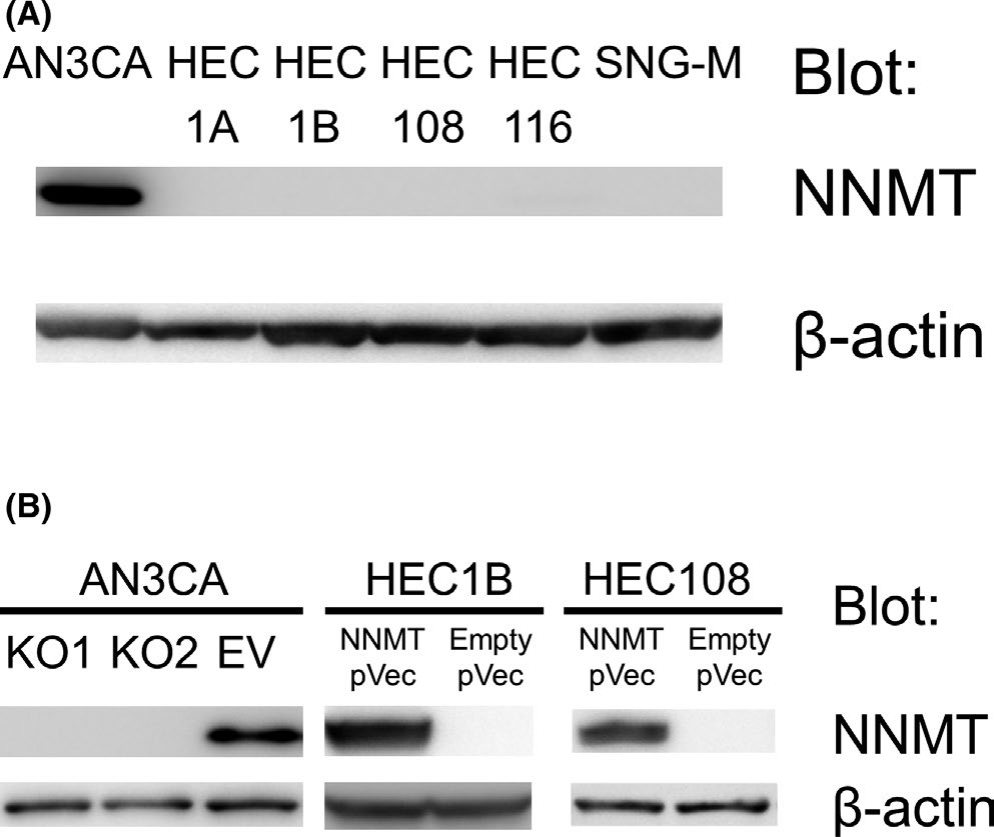
Immunoblotting of NNMT in EC cells and generation of NNMT knockout AN3CA and NNMT‐expressing HEC1B and HEC108. (A) NNMT protein levels in AN3CA, HEC1A, HEC1B, HEC108, HEC116, and SNG‐M. (B) Confirmation of depletion of NNMT in NNMT knockout AN3CA cells (KO1 and KO2) and enrichment of NNMT expression in NNMT‐expressing HEC1B and HEC108 cells by immunoblotting. EV, empty vector control cells

We assessed NNMT expression in AN3CA, HEC1A, HEC1B, HEC108, HEC116, and SNG‐M. NNMT expression was detected only in AN3CA cells (Figure 3A). We assessed the function of NNMT by constructing NNMT knockout AN3CA cells and NNMT‐expressing HEC1B and HEC108 cells.

### 3.3 NNMT promoted the migration, invasion, colony formation, and epithelial–mesenchymal transition of EC

**FIGURE 4:**
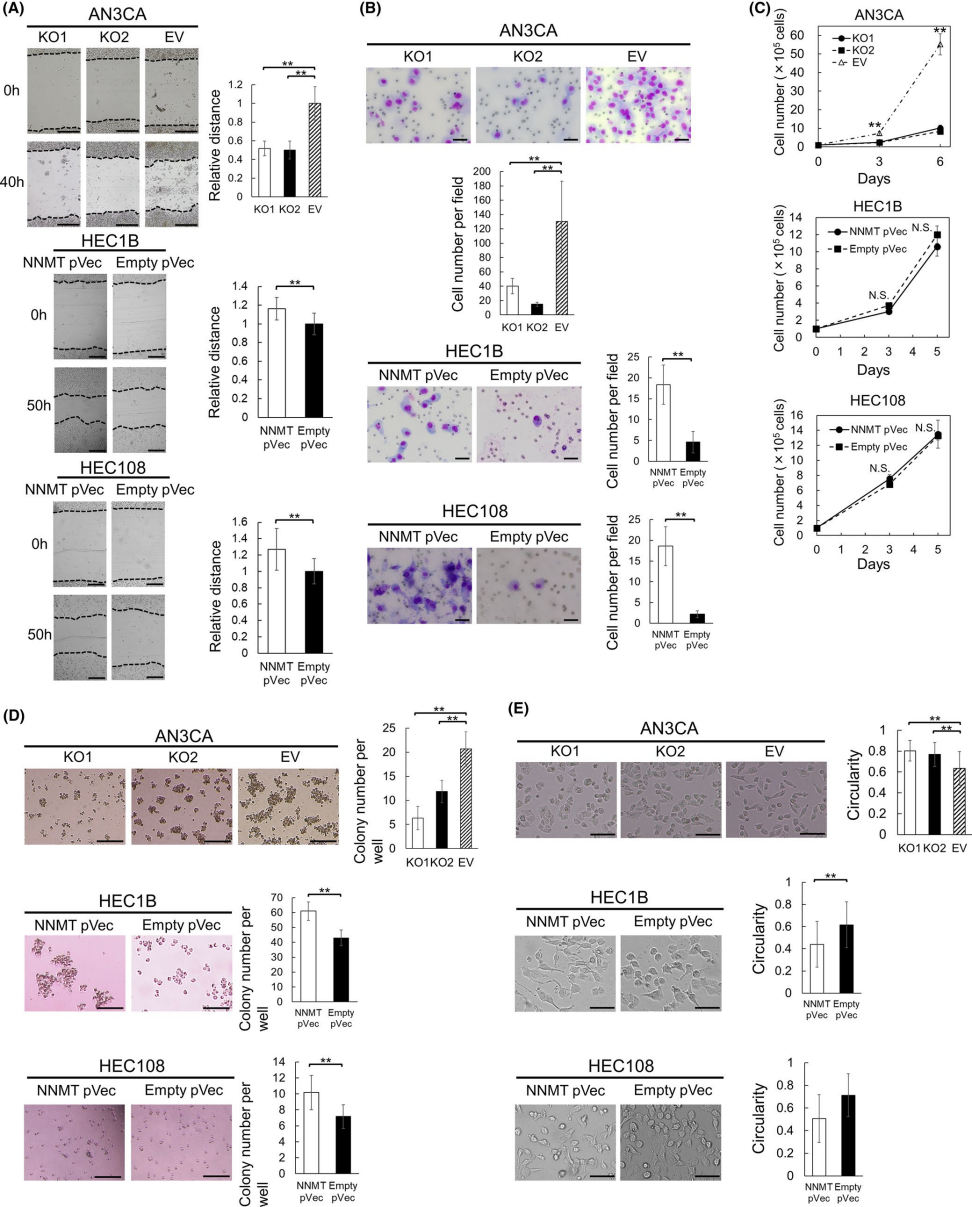
Functional analyses of NNMT. (A) Migration assay. The migration distance was obtained by dividing the width of the wound at 40 h (AN3CA) or 50 h (HEC1B and HEC108) by that at 0 h. The migration distance of control cells (EV or Empty pVec) is expressed as 1. The relative migration distance means a ratio to that of control cells. (B) Matrigel invasion assay. Invading cells are shown in images. We counted invasive cells in five random fields per well. (C) Proliferation assay. (D) Colony formation assay. Colonies were counted per well. (E) Cell morphology. Data are representative of three (AN3CA and HEC1B) or two (HEC108) independent experiments and are shown as means ± SEs. Asterisks mean significant differences, which are determined by the Dunnett's test (AN3CA) and the Student's *t‐*test (HEC1B and HEC108) (*p < 0.05, **p < 0.01). Scale bars: 500 µm in (A), 50 μm in (B), 200 µm in (D), and 100 µm in (E). N.S., not significant

We constructed NNMT knockout AN3CA cell lines and an NNMT‐expressing HEC1B and HEC108 cell line to analyze the function of NNMT (Figure 3B). NNMT depletion attenuated the migration and invasion, while NNMT enrichment had an enhancing effect on migration and invasion (Figure 4A,B). NNMT knockout AN3CA cells showed significantly impaired proliferation and colony formation. On the other hand, NNMT‐expressing HEC1B cells did not show marked changes in proliferation, but formed more colonies (Figure 4C,D). Thus, NNMT is involved in proliferation, not in a two‐dimensional system, but in a three‐dimensional system. Moreover, NNMT knockout cells had a rounded shape and NNMT‐expressing cells had an elongated shape (Figure 4E). We assumed that it may be involved in the EMT.

### 3.4 NNMT activated ERK‐dependent signaling

**FIGURE 5:**
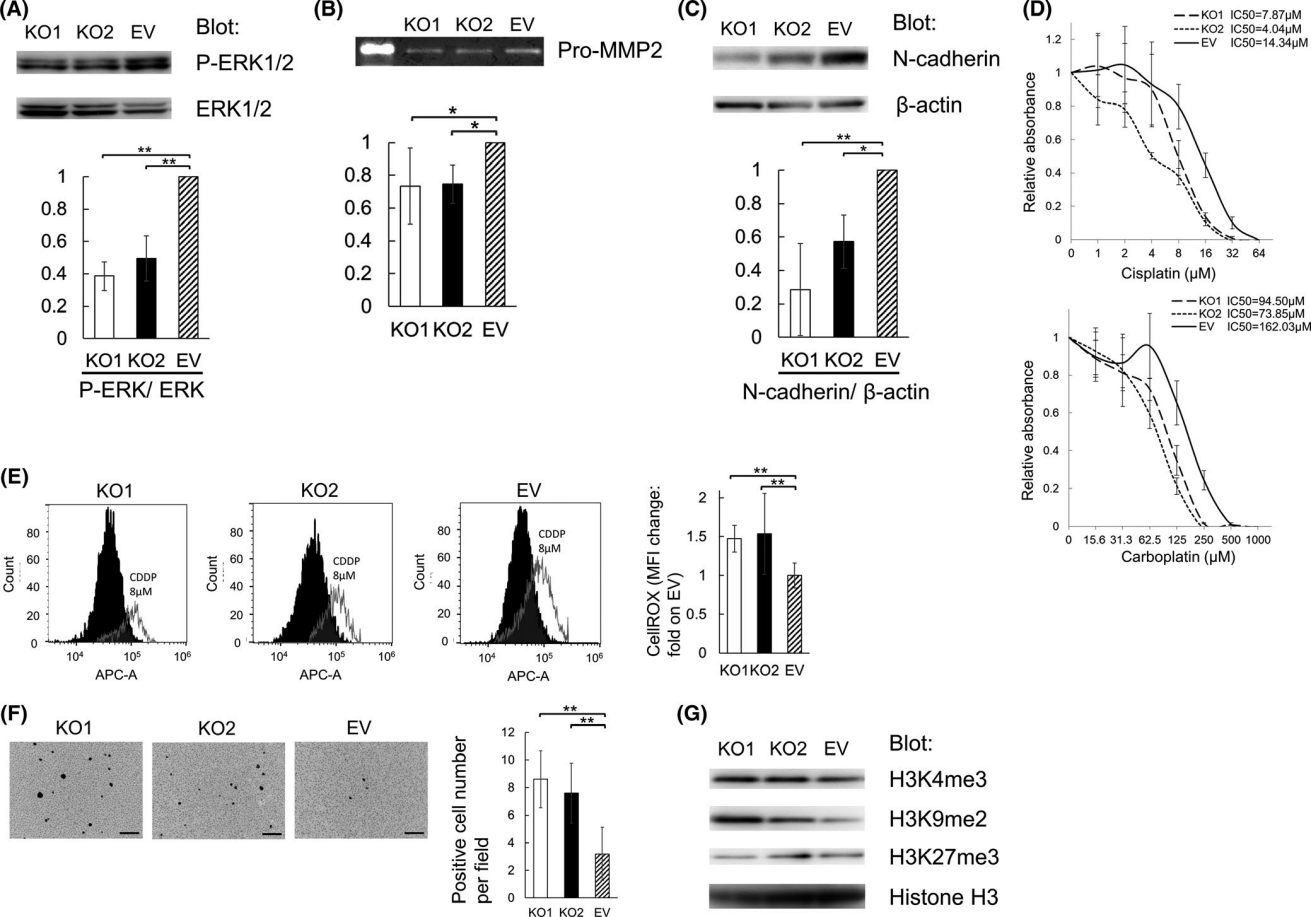
NNMT regulates ERK phosphorylation, MMP2 secretion, N‐cadherin expression, chemoresistance, and H3K9me2 methylation. (A) Immunoblotting of ERK1/2 and phospho‐ERK1/2 protein expression in KO1, KO2, and EV. (B) The expression of pro‐MMP2 in the supernatants obtained KO1, KO2, and EV cells. It was evaluated by gelatin zymography. Equal total protein loading was confirmed using a BCA Protein Assay Kit. The results were quantified using ImageJ. (C) Immunoblotting of N‐cadherin expression in KO1, KO2, and EV cells. (D) Cell viability was compared in the presence of cisplatin or carboplatin. The value of 0 μM is expressed as 1. The relative values of various concentrations of cisplatin (1, 2, 4, 8, 16, 32, or 64 μM) and carboplatin (15.6, 31.3, 62.5, 125, 250, 500, or 1000 μM) are presented as ratios relative to 0 μM. (E) Cellular ROS levels in KO1, KO2, and EV cells. We calculated the change in median fluorescence intensity (MFI) by adding cisplatin and compared the results between KO1, KO2, and EV cells. The change in MFI in EV cells is expressed as 1. (F) Representative images of immunohistochemically staining for cleaved caspase‐3 using cell blocks. Positive cells were counted in five random fields per slide. (G) Immunoblotting to determine H3K4me3, H3K9me2, and H3K27me3 protein levels in KO1, KO2, and EV cells. Equal protein loading was confirmed by quantifying histone H3. Data are representative of three independent experiments and are shown as means ± SEs. Asterisks indicate significant differences as determined by the Dunnett's test (*p < 0.05, **p < 0.01). Scale bars: 100 µm in (F)

ERK1/2 is related to aggressiveness of many types of cancers. We speculated that NNMT may promote carcinogenicity through activation of this signal. Immunoblotting showed that the levels of ERK1/2 phosphorylation were lower in NNMT knockout cells (Figure 5A). This indicated that NNMT promotes malignant potential through ERK1/2 phosphorylation.

### 3.5 Effects of NNMT on MMP2

We investigated the mechanism how NNMT promotes the invasion of EC cells. Matrix metalloproteinases (MMPs) can decompose almost all components of the extracellular matrix.^11^ In MMPs, MMP2 is the main proteolytic enzyme. We speculated that NNMT may activate MMP2 and promote invasion. In gelatin zymography, the expression of pro‐MMP2 in the supernatant of NNMT knockout cells was attenuated (Figure 5B). We found that NNMT enhances invasion by secreting MMP2.

### 3.6 Effects of NNMT on N‐cadherin

The expression of N‐cadherin is known to be elevated by the EMT.^12^ Immunoblotting analyses showed that the expression of N‐cadherin was markedly reduced in NNMT knockout cells (Figure 5C), suggesting that NNMT promotes the EMT.

### 3.7 NNMT knockout attenuates anticancer drug resistance via the production of reactive oxygen species and augments apoptosis

We assessed the anticancer drug effect of NNMT knockout cells. We used cisplatin and carboplatin, as they are commonly used clinically for the treatment of EC. NNMT knockout cells showed augmented cytotoxicity mediated by cisplatin and carboplatin (Figure 5D). Next, we evaluated levels of cellular reactive oxygen species (ROS) in NNMT knockout cells using CellROX Deep Red. NNMT knockout cells showed a greater increase in cellular ROS level with the addition of cisplatin than control cells (Figure 5E). Moreover, we evaluated the apoptosis of NNMT knockout cells. Cleavage of caspase‐3 is generally considered a universal marker of apoptosis. Immunohistochemical staining of cell blocks revealed that the number of cleaved caspase‐3‐positive apoptotic cells in NNMT knockout cells was increased (Figure 5F). Therefore, NNMT was suggested to promote cell survival in response to chemotherapeutic agents by controlling the production of ROS and suppression of apoptosis.

### 3.8 NNMT promotes the methylation of H3K9me2

NNMT depletes S‐adenosyl methionine (SAM), which is a major methyl donor for the methylation of DNA and histones.^13^ We hypothesized that the depletion of NNMT promoted histone methylation. Immunoblotting analyses indicated that the expression of H3K9me2 was enhanced in NNMT knockout cells, while there were no marked changes in the expression of H3K4me3 or H3K27me3 (Figure 5G).

### 3.9 NNMT knockout influences various genes related to migration and invasion

For further analyses of the influence of NNMT knockout, we performed RNA sequencing of NNMT knockout and control AN3CA cells. We analyzed Diseases and Bio Functions via IPA. As shown in Table 2, functions related to cell migration and invasion ranked highly in the analyses. These results indicate that depletion of NNMT influences the expression of various genes related to migration and invasion.

**TABLE 2:** List of diseases and bio functions analyzed by IPA

Name of diseases and bio functions	Activation z‐score^a^
Cell movement	−3.684
Cell movement of tumor cell lines	−3.666
Migration of cells	−3.379
Cell–cell contact	−3.342
Quantity of cells	−3.07
Invasion of tumor cell lines	−2.734
Sprouting	−2.580
Cell spreading	−2.403
Cell spreading of tumor cell lines	−2.384
Proliferation of embryonic cells	−2.032

^a^ Top 10 pathways, with score of less than −2, out of total 846 pathways analyzed by IPA. We took the average of activation z‐score of KO1 versus EV and KO2 versus EV.

## 4 DISCUSSION

We found that NNMT is related to MELF pattern invasion of EC and that depletion of NNMT markedly attenuates the migration and invasion of EC cells. Previously, we reported that high S100A4 and SDPR expression in EC are related to MELF pattern invasion and showed the genetic characteristics of EC with MELF invasion.^3^, ^4^ However, we did not explore what contributed to the characteristic morphology in invasive front area of MELF. Thus, we investigate the molecular features of the invasive front area of EC with the MELF pattern.

To analyze the mechanism of MELF pattern invasion, we performed laser microdissection RNA sequencing analyses and found that sirtuin signaling is related to MELF pattern invasion based on the results of IPA. The sirtuin signaling is known to be regulated by NNMT, not only in normal organs, but also in various tumor tissues.^6^, ^8^, ^9^, ^10^ Therefore, we focused on NNMT. Immunohistochemical analyses showed that the level of NNMT expression was high in the invasive front area of the MELF pattern. This tendency was not found in EC without MELF. Moreover, using EC cell lines, we performed cell function assays. NNMT expression was detected only in AN3CA cells. HEC1A, HEC1B, HEC108, and HEC116 are derived from uterus. On the other hand, AN3CA and SNG‐M are derived from lymph node metastasis. This supports that NNMT is related to lymph node metastasis, which is one of the characteristics of MELF. We showed that NNMT promotes migration, invasion, colony formation, EMT, and chemoresistance. NNMT consumes SAM, which is an essential methylation cofactor. We speculated that the depletion of NNMT promotes histone methylation and leads to tumor suppression. In cell lines, NNMT was related to histone methylation at H3K4, H3K9, and H3K27 in histone H3.^14^ We examined these methylation patterns and showed that the expression of H3K9me2 was enhanced in NNMT knockout cells. Generally, H3K4 methylation is considered to indicate active transcription, whereas H3K9 and H3K27 methylation are associated with transcriptional repression.^15^ We speculated that methylation of H3K9 leads to repression of the transcription of various oncogenic genes. The results of RNA sequencing analyses using NNMT knockout AN3CA also indicated the suppression of various pathways related to cell migration and invasion (Table 2). We speculated that in the invasive front area of MELF, the elevated expression of NNMT might contribute to the characteristic morphology through the suppression of H3K9me2 followed by the enhancement of tumor migration and invasion.

In EC, most cases of G1 have a good prognosis. However, G1 with MELF is more aggressive. MELF is associated with poor prognostic factors, such as lymphovascular invasion and lymph node metastasis. The first choice of treatment for G1 EC is surgical resection. However, G1 MELF has a high risk for recurrence, and different treatment options should be considered in the event of recurrence. Several NNMT inhibitors, which are expected to be useful for the treatment of metabolic disorders, have been developed and are available commercially.^16^ Our findings suggest that NNMT inhibitors may be effective for the treatment of EC. Moreover, a new biosensor was recently developed for rapid quantification of 1‐MNA, which is a metabolite produced by NNMT.^17^ This biosensor may be useful for the development of specific NNMT inhibitors.

Our results indicate that NNMT is related to MELF pattern invasion in EC and promotes migration and invasion via suppression of H3K9me2 methylation. This is the first report of gene analyses focused on the morphological changes in MELF pattern invasion of EC.

## CONFLICT OF INTEREST

All authors have no conflict of interest.

## AUTHORS CONTRIBUTION

S.T., S.N., K.O., and E.M. conceived experiments; S.T. carried out experiments; S.T., Y.H., K.S., M.K., and T.M. collected data; S.T. and D.O. analyzed data; S.T. and E.M. wrote the paper; S.T. generated the figures; All authors agreed to the manuscript content and its submission.

## ETHICS STATEMENT

The present study was approved by the Ethical Review Board of the Graduate School of Medicine, Osaka University (No. 15234).

## Supporting information

Table S1

Table S2

Table S3

Table S4

## Data Availability

The data that support the findings of this study are available from the corresponding author upon reasonable request.
