# Supplementary material for: Nicotinamide N‐methyltransferase is related to MELF pattern invasion in endometrioid carcinoma
Source: Cancer Med. 2021 Oct 16;10(23):8630–40. doi: 10.1002/cam4.4359 (PMC8633241; doi:10.1002/cam4.4359)
Supplement: Supplementary file 1 — Table S1 [file CAM4-10-8630-s003.docx]

**Table S1.** The list of 110 genes showing significantly higher expression in the invasive front area (fold change > 5 and FPKM of invasive front area > 10 in both cases)

| Gene symbol | Gene name |
| --- | --- |
| ADD3-AS1 | ADD3 antisense RNA 1 |
| ALDH1A3 | aldehyde dehydrogenase 1 family member A3 |
| ANKRD37 | ankyrin repeat domain 37 |
| ANKRD53 | ankyrin repeat domain 53 |
| ANKRD9 | ankyrin repeat domain 9 |
| AOC3 | "amine oxidase, copper containing 3" |
| ARPC4-TTLL3 | ARPC4-TTLL3 readthrough |
| AS3MT | arsenite methyltransferase |
| ATG10 | autophagy related 10 |
| BOC | "BOC cell adhesion associated, oncogene regulated" |
| C10orf11 | chromosome 10 open reading frame 11 |
| C16orf96 | chromosome 16 open reading frame 96 |
| C1QTNF1 | C1q and TNF related 1 |
| C5orf46 | chromosome 5 open reading frame 46 |
| CACNA1C | calcium voltage-gated channel subunit alpha1 C |
| CACNA1H | calcium voltage-gated channel subunit alpha1 H |
| CCL14 | C-C motif chemokine ligand 14 |
| CCL17 | C-C motif chemokine ligand 17 |
| CCL18 | C-C motif chemokine ligand 18 |
| CDKN2B-AS1 | CDKN2B antisense RNA 1 |
| CDKN2C | cyclin dependent kinase inhibitor 2C |
| CELF2-AS1 | CELF2 antisense RNA 1 |
| CES1 | carboxylesterase 1 |
| CNN1 | calponin 1 |
| COL16A1 | collagen type XVI alpha 1 chain |
| COL4A2-AS1 | COL4A2 antisense RNA 1 |
| CXCL12 | C-X-C motif chemokine ligand 12 |
| DACT1 | dishevelled binding antagonist of beta catenin 1 |
| DERL3 | derlin 3 |
| DES | desmin |
| DMC1 | DNA meiotic recombinase 1 |
| DOK2 | docking protein 2 |
| DPP6 | dipeptidyl peptidase like 6 |
| ELMO1 | engulfment and cell motility 1 |
| ELN | elastin |
| ERCC6-PGBD3 | ERCC6-PGBD3 readthrough |
| EVA1C | eva-1 homolog C |
| FAM131B | family with sequence similarity 131 member B |
| FAM216A | family with sequence similarity 216 member A |
| FAM83A-AS1 | FAM83A antisense RNA 1 |
| FAM92B | family with sequence similarity 92 member B |
| FBLN5 | fibulin 5 |
| FLNC | filamin C |
| FXYD1 | FXYD domain containing ion transport regulator 1 |
| FXYD6 | FXYD domain containing ion transport regulator 6 |
| GABRR2 | gamma-aminobutyric acid type A receptor rho2 subunit |
| GATA3 | GATA binding protein 3 |
| GEM | GTP binding protein overexpressed in skeletal muscle |
| GNLY | granulysin |
| GPR21 | G protein-coupled receptor 21 |
| HABP4 | hyaluronan binding protein 4 |
| HK3 | hexokinase 3 |
| HSPB7 | heat shock protein family B (small) member 7 |
| IFFO1 | intermediate filament family orphan 1 |
| IGFL2 | IGF like family member 2 |
| IGFN1 | immunoglobulin-like and fibronectin type III domain containing 1 |
| IPO9-AS1 | IPO9 antisense RNA 1 |
| ITGB1BP2 | integrin subunit beta 1 binding protein 2 |
| ITM2A | integral membrane protein 2A |
| KRT14 | keratin 14 |
| LDB3 | LIM domain binding 3 |
| LRRC6 | leucine rich repeat containing 6 |
| LST1 | leukocyte specific transcript 1 |
| LTB4R2 | leukotriene B4 receptor 2 |
| MAP3K10 | mitogen-activated protein kinase kinase kinase 10 |
| MAPK11 | mitogen-activated protein kinase 11 |
| MAPK12 | mitogen-activated protein kinase 12 |
| MARCO | macrophage receptor with collagenous structure |
| METTL18 | methyltransferase like 18 |
| MKLN1-AS | MKLN1 antisense RNA |
| MRGPRF | MAS related GPR family member F |
| MRPS17 | mitochondrial ribosomal protein S17 |
| MSC | musculin |
| NFASC | neurofascin |
| OLFM1 | olfactomedin 1 |
| P2RY6 | pyrimidinergic receptor P2Y6 |
| PDZRN3 | PDZ domain containing ring finger 3 |
| PELI3 | pellino E3 ubiquitin protein ligase family member 3 |
| PHYHIP | phytanoyl-CoA 2-hydroxylase interacting protein |
| PROS1 | protein S |
| PTCD1 | pentatricopeptide repeat domain 1 |
| PTP4A3 | "protein tyrosine phosphatase type IVA, member 3" |
| PWAR4 | Prader Willi/Angelman region RNA 4 |
| RENBP | renin binding protein |
| RETN | resistin |
| RGN | regucalcin |
| RGS11 | regulator of G protein signaling 11 |
| RUVBL1-AS1 | RUVBL1 antisense RNA 1 |
| SAMD14 | sterile alpha motif domain containing 14 |
| SCPEP1 | serine carboxypeptidase 1 |
| SFRP2 | secreted frizzled related protein 2 |
| SH2D3C | SH2 domain containing 3C |
| SHF | Src homology 2 domain containing F |
| SHISA4 | shisa family member 4 |
| SIRPG | signal regulatory protein gamma |
| SLAMF8 | SLAM family member 8 |
| SLC30A3 | solute carrier family 30 member 3 |
| SNAI3-AS1 | SNAI3 antisense RNA 1 |
| SNHG22 | small nucleolar RNA host gene 22 |
| SPEG | SPEG complex locus |
| TAS2R13 | taste 2 receptor member 13 |
| TMEM119 | transmembrane protein 119 |
| TMEM255B | transmembrane protein 255B |
| TRAF3IP3 | TRAF3 interacting protein 3 |
| TRPV2 | transient receptor potential cation channel subfamily V member 2 |
| TSPAN18 | tetraspanin 18 |
| UBASH3A | ubiquitin associated and SH3 domain containing A |
| WNT4 | Wnt family member 4 |
| ZASP | ZO-2 associated speckle protein |
| ZBP1 | Z-DNA binding protein 1 |
