# Supplementary material for: Nicotinamide N‐methyltransferase is related to MELF pattern invasion in endometrioid carcinoma
Source: Cancer Med. 2021 Oct 16;10(23):8630–40. doi: 10.1002/cam4.4359 (PMC8633241; doi:10.1002/cam4.4359)
Supplement: Supplementary file 2 — Table S2 [file CAM4-10-8630-s004.docx]

**Table S2.** The list of 96 genes showing significantly lower expression in the invasive front area (fold change < –5 and FPKM of surface area > 10 in both cases)

| Gene symbol | Gene name |
| --- | --- |
| AGR2 | "anterior gradient 2, protein disulphide isomerase family member" |
| AP1M2 | adaptor related protein complex 1 mu 2 subunit |
| ARL1 | ADP ribosylation factor like GTPase 1 |
| ASRGL1 | asparaginase like 1 |
| ATP1B1 | ATPase Na+/K+ transporting subunit beta 1 |
| BLVRB | biliverdin reductase B |
| BMI1 | "BMI1 proto-oncogene, polycomb ring finger" |
| BRI3BP | BRI3 binding protein |
| C12orf75 | chromosome 12 open reading frame 75 |
| C19orf33 | chromosome 19 open reading frame 33 |
| C1orf186 | chromosome 1 open reading frame 186 |
| C1orf50 | chromosome 1 open reading frame 50 |
| CARD16 | caspase recruitment domain family member 16 |
| CCNB1IP1 | cyclin B1 interacting protein 1 |
| CDH1 | cadherin 1 |
| CFD | complement factor D |
| CKS2 | CDC28 protein kinase regulatory subunit 2 |
| CLPX | caseinolytic mitochondrial matrix peptidase chaperone subunit |
| COMMD1 | copper metabolism domain containing 1 |
| CRYZ | crystallin zeta |
| CYB5R2 | cytochrome b5 reductase 2 |
| DCXR | dicarbonyl and L-xylulose reductase |
| EIF2B1 | eukaryotic translation initiation factor 2B subunit alpha |
| EIF3H | eukaryotic translation initiation factor 3 subunit H |
| EMC2 | ER membrane protein complex subunit 2 |
| ESRP1 | epithelial splicing regulatory protein 1 |
| EXD2 | exonuclease 3'-5' domain containing 2 |
| EZR-AS1 | EZR antisense RNA 1 |
| FAR1 | fatty acyl-CoA reductase 1 |
| FNBP1L | formin binding protein 1 like |
| FOXJ1 | forkhead box J1 |
| FTSJ1 | FtsJ RNA methyltransferase homolog 1 |
| G6PC3 | glucose-6-phosphatase catalytic subunit 3 |
| GALNT7 | polypeptide N-acetylgalactosaminyltransferase 7 |
| GLRX5 | glutaredoxin 5 |
| HIST1H4F | histone cluster 1 H4 family member f |
| HSPB11 | heat shock protein family B (small) member 11 |
| IDH1 | "isocitrate dehydrogenase (NADP(+)) 1, cytosolic" |
| IMP3 | "IMP3, U3 small nucleolar ribonucleoprotein" |
| IMPA2 | inositol monophosphatase 2 |
| ISY1-RAB43 | ISY1-RAB43 readthrough |
| KAT8 | lysine acetyltransferase 8 |
| KLK11 | kallikrein related peptidase 11 |
| KRAS | "KRAS proto-oncogene, GTPase" |
| LAMTOR5 | "late endosomal/lysosomal adaptor, MAPK and MTOR activator 5" |
| LCN12 | lipocalin 12 |
| LGR4 | leucine rich repeat containing G protein-coupled receptor 4 |
| LGR5 | leucine rich repeat containing G protein-coupled receptor 5 |
| MAD2L1 | mitotic arrest deficient 2 like 1 |
| MAP2K6 | mitogen-activated protein kinase kinase 6 |
| MS4A6A | membrane spanning 4-domains A6A |
| NDUFB1 | NADH:ubiquinone oxidoreductase subunit B1 |
| NDUFB5 | NADH:ubiquinone oxidoreductase subunit B5 |
| NDUFS5 | NADH:ubiquinone oxidoreductase subunit S5 |
| NDUFV2 | NADH:ubiquinone oxidoreductase core subunit V2 |
| NFU1 | NFU1 iron-sulfur cluster scaffold |
| NME1 | NME/NM23 nucleoside diphosphate kinase 1 |
| NME2 | NME/NM23 nucleoside diphosphate kinase 2 |
| NOXA1 | NADPH oxidase activator 1 |
| NUDT3 | nudix hydrolase 3 |
| PDCD10 | programmed cell death 10 |
| PHF5A | PHD finger protein 5A |
| PSAT1 | phosphoserine aminotransferase 1 |
| PSMA2 | proteasome subunit alpha 2 |
| RPL36A-HNRNPH2 | RPL36A-HNRNPH2 readthrough |
| SBDS | "SBDS, ribosome maturation factor" |
| SCARNA14 | small Cajal body-specific RNA 14 |
| SCARNA20 | small Cajal body-specific RNA 20 |
| SCFD1 | sec1 family domain containing 1 |
| SCGB1D2 | secretoglobin family 1D member 2 |
| SCGB2A1 | secretoglobin family 2A member 1 |
| SEC11A | "SEC11 homolog A, signal peptidase complex subunit" |
| SGPP2 | sphingosine-1-phosphate phosphatase 2 |
| SHFM1 | split hand/foot malformation (ectrodactyly) type 1 |
| SLC35B1 | solute carrier family 35 member B1 |
| SLC40A1 | solute carrier family 40 member 1 |
| SLPI | secretory leukocyte peptidase inhibitor |
| SNHG25 | small nucleolar RNA host gene 25 |
| SNHG9 | small nucleolar RNA host gene 9 |
| SORT1 | sortilin 1 |
| TIMM23 | translocase of inner mitochondrial membrane 23 |
| TIMM9 | translocase of inner mitochondrial membrane 9 |
| TOMM20 | translocase of outer mitochondrial membrane 20 |
| TPD52L1 | tumor protein D52 like 1 |
| TRIAP1 | TP53 regulated inhibitor of apoptosis 1 |
| TRPT1 | tRNA phosphotransferase 1 |
| UBA52 | ubiquitin A-52 residue ribosomal protein fusion product 1 |
| UBE2A | ubiquitin conjugating enzyme E2 A |
| UBE2V2 | ubiquitin conjugating enzyme E2 V2 |
| USMG5 | up-regulated during skeletal muscle growth 5 homolog (mouse) |
| UXT-AS1 | UXT antisense RNA 1 |
| VTRNA1-2 | vault RNA 1-2 |
| VTRNA1-3 | vault RNA 1-3 |
| ZNF32-AS2 | ZNF32 antisense RNA 2 |
| ZNF622 | zinc finger protein 622 |
| ZNF688 | zinc finger protein 688 |
