# Supplementary material for: Nicotinamide N‐methyltransferase is related to MELF pattern invasion in endometrioid carcinoma
Source: Cancer Med. 2021 Oct 16;10(23):8630–40. doi: 10.1002/cam4.4359 (PMC8633241; doi:10.1002/cam4.4359)
Supplement: Supplementary file 3 — Table S3 [file CAM4-10-8630-s001.docx]

**Table S3.** The list of 345 genes with significantly higher expression in EV than KO1 (fold change < –2 and FPKM of EV > 2)

| Gene symbol | Gene name |
| --- | --- |
| AADAT | aminoadipate aminotransferase |
| ABCD1 | ATP binding cassette subfamily D member 1 |
| ACADM | acyl-CoA dehydrogenase medium chain |
| ACRBP | acrosin binding protein |
| ACY1 | aminoacylase 1 |
| ADI1 | acireductone dioxygenase 1 |
| AIG1 | androgen induced 1 |
| AKR1C2 | aldo-keto reductase family 1 member C2 |
| ANKS1B | ankyrin repeat and sterile alpha motif domain containing 1B |
| ADM5 | adrenomedullin 5 (putative) |
| ANXA2R | annexin A2 receptor |
| AP5S1 | adaptor related protein complex 5 sigma 1 subunit |
| APOC1 | apolipoprotein C1 |
| ARHGAP4 | Rho GTPase activating protein 4 |
| ANTXR1 | anthrax toxin receptor 1 |
| ARMC12 | armadillo repeat containing 12 |
| B4GAT1 | "beta-1,4-glucuronyltransferase 1" |
| B9D2 | B9 domain containing 2 |
| ATP1A1 | ATPase Na+/K+ transporting subunit alpha 1 |
| ATP1A1-AS1 | ATP1A1 antisense RNA 1 |
| BCAP29 | B cell receptor associated protein 29 |
| BEND7 | BEN domain containing 7 |
| BOC | "BOC cell adhesion associated, oncogene regulated" |
| C14orf37 | chromosome 14 open reading frame 37 |
| C16orf86 | chromosome 16 open reading frame 86 |
| C19orf60 | chromosome 19 open reading frame 60 |
| C6orf120 | chromosome 6 open reading frame 120 |
| C6orf52 | chromosome 6 open reading frame 52 |
| ATXN7L2 | ataxin 7 like 2 |
| CAMK2N1 | calcium/calmodulin dependent protein kinase II inhibitor 1 |
| B3GAT2 | "beta-1,3-glucuronyltransferase 2" |
| CCL5 | C-C motif chemokine ligand 5 |
| CCT6B | chaperonin containing TCP1 subunit 6B |
| CD58 | CD58 molecule |
| BAMBI | BMP and activin membrane bound inhibitor |
| BBC3 | BCL2 binding component 3 |
| CDK2AP1 | cyclin dependent kinase 2 associated protein 1 |
| CDRT4 | CMT1A duplicated region transcript 4 |
| CECR5-AS1 | CECR5 antisense RNA 1 |
| BCAN | brevican |
| CENPBD1 | CENPB DNA-binding domain containing 1 |
| CFAP44 | cilia and flagella associated protein 44 |
| CHN1 | chimerin 1 |
| BDKRB2 | bradykinin receptor B2 |
| C1QL1 | complement C1q like 1 |
| CLEC11A | C-type lectin domain containing 11A |
| CLGN | calmegin |
| CPE | carboxypeptidase E |
| CPT1C | carnitine palmitoyltransferase 1C |
| CREB3L4 | cAMP responsive element binding protein 3 like 4 |
| CRNDE | colorectal neoplasia differentially expressed |
| CRYGS | crystallin gamma S |
| CSPG5 | chondroitin sulfate proteoglycan 5 |
| CTSK | cathepsin K |
| CACNA1A | calcium voltage-gated channel subunit alpha1 A |
| CAMK4 | calcium/calmodulin dependent protein kinase IV |
| DEFB109P1 | "defensin, beta 109, pseudogene 1" |
| DFNB59 | "deafness, autosomal recessive 59" |
| CCDC80 | coiled-coil domain containing 80 |
| CCR1 | C-C motif chemokine receptor 1 |
| CDC37L1-AS1 | CDC37L1 antisense RNA 1 (head to head) |
| DNMT3B | DNA methyltransferase 3 beta |
| CDCA7L | cell division cycle associated 7 like |
| DPP7 | dipeptidyl peptidase 7 |
| ECH1 | enoyl-CoA hydratase 1 |
| CDHR1 | cadherin related family member 1 |
| CELSR3-AS1 | CELSR3 antisense RNA 1 (head to head) |
| CHDH | choline dehydrogenase |
| EMX2 | empty spiracles homeobox 2 |
| CHI3L2 | chitinase 3 like 2 |
| CHRNA7 | cholinergic receptor nicotinic alpha 7 subunit |
| ERICH6-AS1 | ERICH6 antisense RNA 1 |
| ERMARD | ER membrane associated RNA degradation |
| ESAM | endothelial cell adhesion molecule |
| CITED4 | Cbp/p300 interacting transactivator with Glu/Asp rich carboxy-terminal domain 4 |
| CKLF-CMTM1 | CKLF-CMTM1 readthrough |
| FAM229A | family with sequence similarity 229 member A |
| CREB5 | cAMP responsive element binding protein 5 |
| FCGR2A | Fc fragment of IgG receptor IIa |
| FCGR2B | Fc fragment of IgG receptor IIb |
| FGFR1 | fibroblast growth factor receptor 1 |
| FHAD1 | forkhead associated phosphopeptide binding domain 1 |
| FHL2 | four and a half LIM domains 2 |
| CRYAB | crystallin alpha B |
| FN1 | fibronectin 1 |
| CTGF | connective tissue growth factor |
| CX3CL1 | C-X3-C motif chemokine ligand 1 |
| FSCN1 | fascin actin-bundling protein 1 |
| FUCA2 | alpha-L-fucosidase 2 |
| CXorf57 | chromosome X open reading frame 57 |
| FZD1 | frizzled class receptor 1 |
| G6PC3 | glucose-6-phosphatase catalytic subunit 3 |
| GAA | "glucosidase alpha, acid" |
| GBA | glucosylceramidase beta |
| GCNT2 | "glucosaminyl (N-acetyl) transferase 2, I-branching enzyme (I blood group)" |
| GDF9 | growth differentiation factor 9 |
| GDPD5 | glycerophosphodiester phosphodiesterase domain containing 5 |
| DHH | desert hedgehog |
| GLMP | glycosylated lysosomal membrane protein |
| GMDS | "GDP-mannose 4,6-dehydratase" |
| DLG4 | discs large MAGUK scaffold protein 4 |
| GNMT | glycine N-methyltransferase |
| GP1BB | glycoprotein Ib platelet beta subunit |
| GRASP | general receptor for phosphoinositides 1 associated scaffold protein |
| GUSB | glucuronidase beta |
| DLL1 | delta like canonical Notch ligand 1 |
| HEXIM2 | hexamethylene bisacetamide inducible 2 |
| DMC1 | DNA meiotic recombinase 1 |
| HIST1H2AG | histone cluster 1 H2A family member g |
| HIST1H2AH | histone cluster 1 H2A family member h |
| HIST1H2BL | histone cluster 1 H2B family member l |
| HIST1H3B | histone cluster 1 H3 family member b |
| HIST1H4H | histone cluster 1 H4 family member h |
| HLA-DMA | "major histocompatibility complex, class II, DM alpha" |
| HLA-DMB | "major histocompatibility complex, class II, DM beta" |
| DNALI1 | dynein axonemal light intermediate chain 1 |
| HLX | H2.0 like homeobox |
| DOK1 | docking protein 1 |
| HOXA-AS2 | HOXA cluster antisense RNA 2 |
| HOXB6 | homeobox B6 |
| HOXC4 | homeobox C4 |
| HOXC5 | homeobox C5 |
| HOXD13 | homeobox D13 |
| HSPA6 | heat shock protein family A (Hsp70) member 6 |
| HSPA7 | heat shock protein family A (Hsp70) member 7 |
| IDI2 | isopentenyl-diphosphate delta isomerase 2 |
| EFNB3 | ephrin B3 |
| EGLN3 | egl-9 family hypoxia inducible factor 3 |
| IFT46 | intraflagellar transport 46 |
| ELFN1-AS1 | ELFN1 antisense RNA 1 |
| IL11RA | interleukin 11 receptor subunit alpha |
| ELOVL4 | ELOVL fatty acid elongase 4 |
| ENC1 | ectodermal-neural cortex 1 |
| INO80B-WBP1 | INO80B-WBP1 readthrough (NMD candidate) |
| IQCH-AS1 | IQCH antisense RNA 1 |
| ITGB1BP2 | integrin subunit beta 1 binding protein 2 |
| KCNIP2 | potassium voltage-gated channel interacting protein 2 |
| KCNMB4 | potassium calcium-activated channel subfamily M regulatory beta subunit 4 |
| KDM8 | lysine demethylase 8 |
| KHDC1 | KH domain containing 1 |
| ENPP1 | ectonucleotide pyrophosphatase/phosphodiesterase 1 |
| KLK2 | kallikrein related peptidase 2 |
| KLRK1 | killer cell lectin like receptor K1 |
| LAT2 | linker for activation of T cells family member 2 |
| LDHC | lactate dehydrogenase C |
| EPHB6 | EPH receptor B6 |
| LINC00310 | long intergenic non-protein coding RNA 310 |
| LINC00969 | MIR570 host gene? |
| LINC01098 | long intergenic non-protein coding RNA 1098 |
| ETV2 | ETS variant 2 |
| LINC01159 | long intergenic non-protein coding RNA 1159 |
| FABP6 | fatty acid binding protein 6 |
| LMF1 | lipase maturation factor 1 |
| LOC100128361 | uncharacterized LOC100128361 |
| FAM107B | family with sequence similarity 107 member B |
| LOC100240735 | uncharacterized LOC100240735 |
| LOC100506457 | uncharacterized LOC100506457 |
| LOC100507006 | uncharacterized LOC100507006 |
| LOC101928069 | uncharacterized LOC101928069 |
| LOC101928100 | uncharacterized LOC101928100 |
| LOC101929089 | uncharacterized LOC101929089 |
| LOC105373383 | uncharacterized LOC105373383 |
| LOC105377348 | uncharacterized LOC105377348 |
| LOC729970 | hCG2028352-like |
| LRFN3 | leucine rich repeat and fibronectin type III domain containing 3 |
| LRFN5 | leucine rich repeat and fibronectin type III domain containing 5 |
| LZTS3 | leucine zipper tumor suppressor family member 3 |
| MACROD1 | MACRO domain containing 1 |
| FAM178B | family with sequence similarity 178 member B |
| MAGEC2 | MAGE family member C2 |
| MAN1B1 | mannosidase alpha class 1B member 1 |
| MANF | mesencephalic astrocyte derived neurotrophic factor |
| MAPK4 | mitogen-activated protein kinase 4 |
| FAM195B | "family with sequence similarity 195, member B" |
| METRN | "meteorin, glial cell differentiation regulator" |
| METTL7B | methyltransferase like 7B |
| MIA | melanoma inhibitory activity |
| MIA-RAB4B | MIA-RAB4B readthrough (NMD candidate) |
| MIR503HG | MIR503 host gene |
| MIR671 | microRNA 671 |
| MMP11 | matrix metallopeptidase 11 |
| MMP25-AS1 | MMP25 antisense RNA 1 |
| MYLK-AS1 | MYLK antisense RNA 1 |
| FAM46A | family with sequence similarity 46 member A |
| FAM71E1 | family with sequence similarity 71 member E1 |
| FLVCR1-AS1 | FLVCR1 antisense RNA 1 (head to head) |
| FNBP1L | formin binding protein 1 like |
| NPB | neuropeptide B |
| NPIPA5 | nuclear pore complex interacting protein family member A5 |
| NUDT4P1 | nudix (nucleoside diphosphate linked moiety X)-type motif 4 pseudogene 1 |
| FRAT1 | "FRAT1, WNT signaling pathway regulator" |
| OLMALINC | oligodendrocyte maturation-associated long intergenic non-coding RNA |
| P3H4 | prolyl 3-hydroxylase family member 4 (non-enzymatic) |
| PARP10 | poly(ADP-ribose) polymerase family member 10 |
| PCDHGC3 | "protocadherin gamma subfamily C, 3" |
| PDCL3P4 | phosducin-like 3 pseudogene 4 |
| PDIA3P1 | protein disulfide isomerase family A member 3 pseudogene 1 |
| PECR | peroxisomal trans-2-enoyl-CoA reductase |
| PGLS | 6-phosphogluconolactonase |
| PHLDA3 | pleckstrin homology like domain family A member 3 |
| FXYD6 | FXYD domain containing ion transport regulator 6 |
| PLAC8L1 | PLAC8 like 1 |
| PLOD1 | "procollagen-lysine,2-oxoglutarate 5-dioxygenase 1" |
| PPT1 | palmitoyl-protein thioesterase 1 |
| PRLR | prolactin receptor |
| PSD | pleckstrin and Sec7 domain containing |
| PSMB1 | proteasome subunit beta 1 |
| PSPN | persephin |
| GDPD3 | glycerophosphodiester phosphodiesterase domain containing 3 |
| RAB3A | "RAB3A, member RAS oncogene family" |
| RAET1G | retinoic acid early transcript 1G |
| GGH | gamma-glutamyl hydrolase |
| REV3L | "REV3 like, DNA directed polymerase zeta catalytic subunit" |
| GJC1 | gap junction protein gamma 1 |
| RHEBL1 | RHEB like 1 |
| GLB1L2 | galactosidase beta 1 like 2 |
| RNASET2 | ribonuclease T2 |
| RNU11 | "RNA, U11 small nuclear" |
| ROM1 | retinal outer segment membrane protein 1 |
| GNG7 | G protein subunit gamma 7 |
| RPS2P32 | ribosomal protein S2 pseudogene 32 |
| HEPH | hephaestin |
| HHLA3 | HERV-H LTR-associating 3 |
| HIST1H1E | histone cluster 1 H1 family member e |
| S100A13 | S100 calcium binding protein A13 |
| S100A4 | S100 calcium binding protein A4 |
| SAPCD1-AS1 | SAPCD1 antisense RNA 1 |
| SARDH | sarcosine dehydrogenase |
| SCARNA5 | small Cajal body-specific RNA 5 |
| HLA-DRB1 | "major histocompatibility complex, class II, DR beta 1" |
| HOGA1 | 4-hydroxy-2-oxoglutarate aldolase 1 |
| HOXB3 | homeobox B3 |
| HOXB5 | homeobox B5 |
| HSPB2 | heat shock protein family B (small) member 2 |
| SLC27A3 | solute carrier family 27 member 3 |
| SLC30A3 | solute carrier family 30 member 3 |
| SLC39A11 | solute carrier family 39 member 11 |
| SMPD2 | sphingomyelin phosphodiesterase 2 |
| SNORA10 | "small nucleolar RNA, H/ACA box 10" |
| SNORA27 | "small nucleolar RNA, H/ACA box 27" |
| SNORA32 | "small nucleolar RNA, H/ACA box 32" |
| IFI27 | interferon alpha inducible protein 27 |
| IFITM1 | interferon induced transmembrane protein 1 |
| IGSF11 | immunoglobulin superfamily member 11 |
| IL13RA2 | interleukin 13 receptor subunit alpha 2 |
| SNORA68 | "small nucleolar RNA, H/ACA box 68" |
| SNORA74A | "small nucleolar RNA, H/ACA box 74A" |
| IL17RB | interleukin 17 receptor B |
| SNX14 | sorting nexin 14 |
| SOX8 | SRY-box 8 |
| SPARC | secreted protein acidic and cysteine rich |
| ST3GAL4-AS1 | G-quadruplex forming sequence containing lncRNA |
| KIF26B | kinesin family member 26B |
| SUV420H2 | suppressor of variegation 4-20 homolog 2 (Drosophila) |
| SYPL2 | synaptophysin like 2 |
| TAGLN2 | transgelin 2 |
| LGALS3BP | galectin 3 binding protein |
| THAP8 | THAP domain containing 8 |
| THOC7 | THO complex 7 |
| THSD4 | thrombospondin type 1 domain containing 4 |
| TIAF1 | TGFB1-induced anti-apoptotic factor 1 |
| TICAM2 | toll like receptor adaptor molecule 2 |
| TMED3 | transmembrane p24 trafficking protein 3 |
| TMEM158 | transmembrane protein 158 (gene/pseudogene) |
| LHPP | phospholysine phosphohistidine inorganic pyrophosphate phosphatase |
| TMEM39B | transmembrane protein 39B |
| LINC00673 | long intergenic non-protein coding RNA 673 |
| LINC01158 | long intergenic non-protein coding RNA 1158 |
| LINCR-0002 | uncharacterized LincR-0002 |
| TRAF3IP2 | TRAF3 interacting protein 2 |
| TREX2 | three prime repair exonuclease 2 |
| LOC100240734 | uncharacterized LOC100240734 |
| LOC101928307 | uncharacterized LOC101928307 |
| UBE3D | ubiquitin protein ligase E3D |
| VAMP8 | vesicle associated membrane protein 8 |
| VAX2 | ventral anterior homeobox 2 |
| ZDHHC2 | zinc finger DHHC-type containing 2 |
| ZGLP1 | "zinc finger, GATA-like protein 1" |
| ZNF414 | zinc finger protein 414 |
| ZNF438 | zinc finger protein 438 |
| ZP3 | zona pellucida glycoprotein 3 |
| MAFA-AS1 | MAFA antisense RNA 1 |
| MARCKS | myristoylated alanine rich protein kinase C substrate |
| MLLT6 | "MLLT6, PHD finger containing" |
| MMP17 | matrix metallopeptidase 17 |
| MYO10 | myosin X |
| MZF1 | myeloid zinc finger 1 |
| NAT6 | N-acetyltransferase 6 |
| NDRG4 | NDRG family member 4 |
| NKX6-1 | NK6 homeobox 1 |
| NLRP4 | NLR family pyrin domain containing 4 |
| NNMT | nicotinamide N-methyltransferase |
| NPAS2 | neuronal PAS domain protein 2 |
| NPFF | neuropeptide FF-amide peptide precursor |
| NUPR1 | "nuclear protein 1, transcriptional regulator" |
| PDE6H | phosphodiesterase 6H |
| PITX2 | paired like homeodomain 2 |
| PLEKHB1 | pleckstrin homology domain containing B1 |
| PRKD3 | protein kinase D3 |
| RAB17 | "RAB17, member RAS oncogene family" |
| REP15 | RAB15 effector protein |
| RGR | retinal G protein coupled receptor |
| RIN3 | Ras and Rab interactor 3 |
| RNF208 | ring finger protein 208 |
| RPH3AL | rabphilin 3A like (without C2 domains) |
| RPLP0P2 | ribosomal protein lateral stalk subunit P0 pseudogene 2 |
| RPS18P9 | ribosomal protein S18 pseudogene 9 |
| RPS6KA2 | ribosomal protein S6 kinase A2 |
| RTKN2 | rhotekin 2 |
| S100A1 | S100 calcium binding protein A1 |
| S100A16 | S100 calcium binding protein A16 |
| SAPCD1 | suppressor APC domain containing 1 |
| SELENBP1 | selenium binding protein 1 |
| SERPINE2 | serpin family E member 2 |
| SFRP1 | secreted frizzled related protein 1 |
| SH3KBP1 | SH3 domain containing kinase binding protein 1 |
| SH3TC1 | SH3 domain and tetratricopeptide repeats 1 |
| SLC16A10 | solute carrier family 16 member 10 |
| SLC1A3 | solute carrier family 1 member 3 |
| SLC29A2 | solute carrier family 29 member 2 |
| SNHG5 | small nucleolar RNA host gene 5 |
| SNORA41 | "small nucleolar RNA, H/ACA box 41" |
| SNORA44 | "small nucleolar RNA, H/ACA box 44" |
| SNORA47 | "small nucleolar RNA, H/ACA box 47" |
| SNORA49 | "small nucleolar RNA, H/ACA box 49" |
| SNORA64 | "small nucleolar RNA, H/ACA box 64" |
| SNORA74B | "small nucleolar RNA, H/ACA box 74B" |
| SNORD15A | "small nucleolar RNA, C/D box 15A" |
| STAC3 | SH3 and cysteine rich domain 3 |
| STARD8 | StAR related lipid transfer domain containing 8 |
| SYT11 | synaptotagmin 11 |
| TEN1-CDK3 | TEN1-CDK3 readthrough (NMD candidate) |
| TGIF2-C20orf24 | TGIF2-C20orf24 readthrough |
| TMEM220-AS1 | TMEM220 antisense RNA 1 |
| TMEM91 | transmembrane protein 91 |
| TMEM98 | transmembrane protein 98 |
| TPD52L1 | tumor protein D52 like 1 |
| TPI1P3 | triosephosphate isomerase 1 pseudogene 3 |
| TPM2 | tropomyosin 2 |
| TSLP | thymic stromal lymphopoietin |
| TTYH1 | tweety family member 1 |
| UBE2E3 | ubiquitin conjugating enzyme E2 E3 |
| UBL3 | ubiquitin like 3 |
| YTHDF3-AS1 | YTHDF3 antisense RNA 1 (head to head) |
| ZNF837 | zinc finger protein 837 |
