# Supplementary material for: Nicotinamide N‐methyltransferase is related to MELF pattern invasion in endometrioid carcinoma
Source: Cancer Med. 2021 Oct 16;10(23):8630–40. doi: 10.1002/cam4.4359 (PMC8633241; doi:10.1002/cam4.4359)
Supplement: Supplementary file 4 — Table S4 [file CAM4-10-8630-s002.docx]

**Table S4.** The list of 289 genes with significantly higher expression in EV than in KO2 (fold change < –2 and FPKM of EV > 2)

| Gene symbol | Gene name |
| --- | --- |
| AADAT | aminoadipate aminotransferase |
| ADAMTS7 | ADAM metallopeptidase with thrombospondin type 1 motif 7 |
| ADM5 | adrenomedullin 5 (putative) |
| AGPAT4 | 1-acylglycerol-3-phosphate O-acyltransferase 4 |
| ANKRD10 | ankyrin repeat domain 10 |
| ANTXR1 | anthrax toxin receptor 1 |
| ARGLU1 | arginine and glutamate rich 1 |
| ARMC12 | armadillo repeat containing 12 |
| ASF1A | anti-silencing function 1A histone chaperone |
| ATP1A1 | ATPase Na+/K+ transporting subunit alpha 1 |
| ATP1A1-AS1 | ATP1A1 antisense RNA 1 |
| ATP2A1-AS1 | ATP2A1 antisense RNA 1 |
| ATP2A3 | ATPase sarcoplasmic/endoplasmic reticulum Ca2+ transporting 3 |
| ATXN7L2 | ataxin 7 like 2 |
| AUTS2 | "AUTS2, activator of transcription and developmental regulator" |
| B3GAT2 | "beta-1,3-glucuronyltransferase 2" |
| B4GALNT4 | "beta-1,4-N-acetyl-galactosaminyltransferase 4" |
| BAMBI | BMP and activin membrane bound inhibitor |
| BBC3 | BCL2 binding component 3 |
| BCAM | basal cell adhesion molecule (Lutheran blood group) |
| BCAN | brevican |
| BCL3 | B cell CLL/lymphoma 3 |
| BCYRN1 | brain cytoplasmic RNA 1 |
| BDKRB2 | bradykinin receptor B2 |
| BRSK1 | BR serine/threonine kinase 1 |
| C1QL1 | complement C1q like 1 |
| C1R | complement C1r |
| C1S | complement C1s |
| C3orf58 | chromosome 3 open reading frame 58 |
| CACNA1A | calcium voltage-gated channel subunit alpha1 A |
| CAMK4 | calcium/calmodulin dependent protein kinase IV |
| CARKD | carbohydrate kinase domain containing |
| CCDC80 | coiled-coil domain containing 80 |
| CCNO | cyclin O |
| CCR1 | C-C motif chemokine receptor 1 |
| CDC37L1-AS1 | CDC37L1 antisense RNA 1 (head to head) |
| CDCA7L | cell division cycle associated 7 like |
| CDHR1 | cadherin related family member 1 |
| CDK6 | cyclin dependent kinase 6 |
| CELSR3-AS1 | CELSR3 antisense RNA 1 (head to head) |
| CHDH | choline dehydrogenase |
| CHI3L2 | chitinase 3 like 2 |
| CHRD | chordin |
| CHRNA7 | cholinergic receptor nicotinic alpha 7 subunit |
| CITED4 | Cbp/p300 interacting transactivator with Glu/Asp rich carboxy-terminal domain 4 |
| CKB | creatine kinase B |
| CKLF-CMTM1 | CKLF-CMTM1 readthrough |
| CLYBL | citrate lyase beta like |
| COL9A2 | collagen type IX alpha 2 chain |
| CPS1 | carbamoyl-phosphate synthase 1 |
| CREB5 | cAMP responsive element binding protein 5 |
| CRYAB | crystallin alpha B |
| CTGF | connective tissue growth factor |
| CX3CL1 | C-X3-C motif chemokine ligand 1 |
| CXorf57 | chromosome X open reading frame 57 |
| CYP2R1 | cytochrome P450 family 2 subfamily R member 1 |
| DHH | desert hedgehog |
| DHRS2 | dehydrogenase/reductase 2 |
| DKKL1 | dickkopf like acrosomal protein 1 |
| DLG4 | discs large MAGUK scaffold protein 4 |
| DLL1 | delta like canonical Notch ligand 1 |
| DMC1 | DNA meiotic recombinase 1 |
| DNAJC12 | DnaJ heat shock protein family (Hsp40) member C12 |
| DNALI1 | dynein axonemal light intermediate chain 1 |
| DOK1 | docking protein 1 |
| ECEL1 | endothelin converting enzyme like 1 |
| ECHDC1 | ethylmalonyl-CoA decarboxylase 1 |
| EEF1E1-BLOC1S5 | EEF1E1-BLOC1S5 readthrough (NMD candidate) |
| EFNB3 | ephrin B3 |
| EGLN3 | egl-9 family hypoxia inducible factor 3 |
| ELFN1-AS1 | ELFN1 antisense RNA 1 |
| ELOVL4 | ELOVL fatty acid elongase 4 |
| ENC1 | ectodermal-neural cortex 1 |
| ENPP1 | ectonucleotide pyrophosphatase/phosphodiesterase 1 |
| EPHB6 | EPH receptor B6 |
| EPM2A | "EPM2A, laforin glucan phosphatase" |
| ERRFI1 | ERBB receptor feedback inhibitor 1 |
| ETV2 | ETS variant 2 |
| FABP6 | fatty acid binding protein 6 |
| FAM107B | family with sequence similarity 107 member B |
| FAM171A1 | family with sequence similarity 171 member A1 |
| FAM178B | family with sequence similarity 178 member B |
| FAM195B | "family with sequence similarity 195, member B" |
| FAM198A | family with sequence similarity 198 member A |
| FAM226B | family with sequence similarity 226 member B (non-protein coding) |
| FAM229B | family with sequence similarity 229 member B |
| FAM46A | family with sequence similarity 46 member A |
| FAM71E1 | family with sequence similarity 71 member E1 |
| FARP1 | "FERM, ARH/RhoGEF and pleckstrin domain protein 1" |
| FGFR4 | fibroblast growth factor receptor 4 |
| FLJ46906 | uncharacterized LOC441172 |
| FLVCR1-AS1 | FLVCR1 antisense RNA 1 (head to head) |
| FNBP1L | formin binding protein 1 like |
| FOXF2 | forkhead box F2 |
| FRAT1 | "FRAT1, WNT signaling pathway regulator" |
| FSD1 | fibronectin type III and SPRY domain containing 1 |
| FXYD6 | FXYD domain containing ion transport regulator 6 |
| GAS6 | growth arrest specific 6 |
| GATA5 | GATA binding protein 5 |
| GDPD3 | glycerophosphodiester phosphodiesterase domain containing 3 |
| GGH | gamma-glutamyl hydrolase |
| GJC1 | gap junction protein gamma 1 |
| GLB1L2 | galactosidase beta 1 like 2 |
| GNG2 | G protein subunit gamma 2 |
| GNG7 | G protein subunit gamma 7 |
| GNRH2 | gonadotropin releasing hormone 2 |
| GTF3C6 | general transcription factor IIIC subunit 6 |
| HEPH | hephaestin |
| HHLA3 | HERV-H LTR-associating 3 |
| HIST1H1E | histone cluster 1 H1 family member e |
| HIST1H2AK | histone cluster 1 H2A family member k |
| HIST1H2BF | histone cluster 1 H2B family member f |
| HIST1H4B | histone cluster 1 H4 family member b |
| HLA-DRB1 | "major histocompatibility complex, class II, DR beta 1" |
| HOGA1 | 4-hydroxy-2-oxoglutarate aldolase 1 |
| HOXB3 | homeobox B3 |
| HOXB5 | homeobox B5 |
| HOXC-AS2 | HOXC cluster antisense RNA 2 |
| HSPB2 | heat shock protein family B (small) member 2 |
| HSPG2 | heparan sulfate proteoglycan 2 |
| HTRA1 | HtrA serine peptidase 1 |
| HYAL3 | hyaluronoglucosaminidase 3 |
| IFI27 | interferon alpha inducible protein 27 |
| IFITM1 | interferon induced transmembrane protein 1 |
| IGSF11 | immunoglobulin superfamily member 11 |
| IL13RA2 | interleukin 13 receptor subunit alpha 2 |
| IL17RB | interleukin 17 receptor B |
| JUP | junction plakoglobin |
| KDELC1 | KDEL motif containing 1 |
| KIF26B | kinesin family member 26B |
| KLF15 | Kruppel like factor 15 |
| LAMP1 | lysosomal associated membrane protein 1 |
| LGALS3BP | galectin 3 binding protein |
| LHPP | phospholysine phosphohistidine inorganic pyrophosphate phosphatase |
| LINC00659 | long intergenic non-protein coding RNA 659 |
| LINC00673 | long intergenic non-protein coding RNA 673 |
| LINC01158 | long intergenic non-protein coding RNA 1158 |
| LINCR-0002 | uncharacterized LincR-0002 |
| LOC100129461 | uncharacterized LOC100129461 |
| LOC100240734 | uncharacterized LOC100240734 |
| LOC101928307 | uncharacterized LOC101928307 |
| LOC103021295 | uncharacterized LOC103021295 |
| LOC730183 | uncharacterized LOC730183 |
| LOC730202 | uncharacterized LOC730202 |
| LY6G5C | lymphocyte antigen 6 family member G5C |
| MAFA-AS1 | MAFA antisense RNA 1 |
| MARCKS | myristoylated alanine rich protein kinase C substrate |
| MEF2B | myocyte enhancer factor 2B |
| MIR1307 | microRNA 1307 |
| MIR431 | microRNA 431 |
| MIR7-1 | microRNA 7-1 |
| MLLT6 | "MLLT6, PHD finger containing" |
| MMP17 | matrix metallopeptidase 17 |
| MT1X | metallothionein 1X |
| MTHFD1L | methylenetetrahydrofolate dehydrogenase (NADP+ dependent) 1 like |
| MYLK2 | myosin light chain kinase 2 |
| MYO10 | myosin X |
| MZF1 | myeloid zinc finger 1 |
| NAB1 | NGFI-A binding protein 1 |
| NAT6 | N-acetyltransferase 6 |
| NDRG4 | NDRG family member 4 |
| NDUFA4L2 | "NDUFA4, mitochondrial complex associated like 2" |
| NELL2 | neural EGFL like 2 |
| NES | nestin |
| NKX6-1 | NK6 homeobox 1 |
| NLRP4 | NLR family pyrin domain containing 4 |
| NMU | neuromedin U |
| NNMT | nicotinamide N-methyltransferase |
| NPAS2 | neuronal PAS domain protein 2 |
| NPFF | neuropeptide FF-amide peptide precursor |
| NR1H3 | nuclear receptor subfamily 1 group H member 3 |
| NRDE2 | "NRDE-2, necessary for RNA interference, domain containing" |
| NUPR1 | "nuclear protein 1, transcriptional regulator" |
| OASL | 2'-5'-oligoadenylate synthetase like |
| ORC3 | origin recognition complex subunit 3 |
| OSBPL9 | oxysterol binding protein like 9 |
| PBX1 | PBX homeobox 1 |
| PDE6H | phosphodiesterase 6H |
| PERP | "PERP, TP53 apoptosis effector" |
| PIK3CD-AS2 | PIK3CD antisense RNA 2 |
| PITX2 | paired like homeodomain 2 |
| PLAT | "plasminogen activator, tissue type" |
| PLEKHB1 | pleckstrin homology domain containing B1 |
| PMP22 | peripheral myelin protein 22 |
| PMS2P2 | "PMS1 homolog 2, mismatch repair system component pseudogene 2" |
| PRKD3 | protein kinase D3 |
| PRR4 | proline rich 4 |
| PRSS23 | serine protease 23 |
| PSTK | phosphoseryl-tRNA kinase |
| PYCARD-AS1 | PYCARD antisense RNA 1 |
| QRFP | pyroglutamylated RFamide peptide |
| RAB17 | "RAB17, member RAS oncogene family" |
| RAB4B | "RAB4B, member RAS oncogene family" |
| RALY-AS1 | RALY antisense RNA 1 |
| RASA3 | RAS p21 protein activator 3 |
| REP15 | RAB15 effector protein |
| RGR | retinal G protein coupled receptor |
| RIN3 | Ras and Rab interactor 3 |
| RMND1 | required for meiotic nuclear division 1 homolog |
| RNF208 | ring finger protein 208 |
| RNF5 | ring finger protein 5 |
| RPH3AL | rabphilin 3A like (without C2 domains) |
| RPL17-C18orf32 | RPL17-C18orf32 readthrough |
| RPLP0P2 | ribosomal protein lateral stalk subunit P0 pseudogene 2 |
| RPS10-NUDT3 | RPS10-NUDT3 readthrough |
| RPS18P9 | ribosomal protein S18 pseudogene 9 |
| RPS6KA2 | ribosomal protein S6 kinase A2 |
| RPSAP9 | ribosomal protein SA pseudogene 9 |
| RRBP1 | ribosome binding protein 1 |
| RTKN2 | rhotekin 2 |
| RTL1 | retrotransposon Gag like 1 |
| S100A1 | S100 calcium binding protein A1 |
| S100A16 | S100 calcium binding protein A16 |
| SAPCD1 | suppressor APC domain containing 1 |
| SCAMP5 | secretory carrier membrane protein 5 |
| SCARNA8 | small Cajal body-specific RNA 8 |
| SCML1 | Scm polycomb group protein like 1 |
| SDHAF4 | succinate dehydrogenase complex assembly factor 4 |
| SEC63 | "SEC63 homolog, protein translocation regulator" |
| SELENBP1 | selenium binding protein 1 |
| SEMA6B | semaphorin 6B |
| SEPT10 | septin 10 |
| SERPINE2 | serpin family E member 2 |
| SERPINH1 | serpin family H member 1 |
| SF3B5 | splicing factor 3b subunit 5 |
| SFRP1 | secreted frizzled related protein 1 |
| SH3BGR | SH3 domain binding glutamate rich protein |
| SH3KBP1 | SH3 domain containing kinase binding protein 1 |
| SH3TC1 | SH3 domain and tetratricopeptide repeats 1 |
| SHC4 | SHC adaptor protein 4 |
| SLC16A10 | solute carrier family 16 member 10 |
| SLC16A4 | solute carrier family 16 member 4 |
| SLC1A3 | solute carrier family 1 member 3 |
| SLC27A1 | solute carrier family 27 member 1 |
| SLC29A2 | solute carrier family 29 member 2 |
| SLC2A1 | solute carrier family 2 member 1 |
| SNHG5 | small nucleolar RNA host gene 5 |
| SNORA20 | "small nucleolar RNA, H/ACA box 20" |
| SNORA25 | "small nucleolar RNA, H/ACA box 25" |
| SNORA29 | "small nucleolar RNA, H/ACA box 29" |
| SNORA41 | "small nucleolar RNA, H/ACA box 41" |
| SNORA43 | "small nucleolar RNA, H/ACA box 43" |
| SNORA44 | "small nucleolar RNA, H/ACA box 44" |
| SNORA47 | "small nucleolar RNA, H/ACA box 47" |
| SNORA48 | "small nucleolar RNA, H/ACA box 48" |
| SNORA49 | "small nucleolar RNA, H/ACA box 49" |
| SNORA52 | "small nucleolar RNA, H/ACA box 52" |
| SNORA57 | "small nucleolar RNA, H/ACA box 57" |
| SNORA5C | "small nucleolar RNA, H/ACA box 5C" |
| SNORA64 | "small nucleolar RNA, H/ACA box 64" |
| SNORA70 | "small nucleolar RNA, H/ACA box 70" |
| SNORA74B | "small nucleolar RNA, H/ACA box 74B" |
| SNORA8 | "small nucleolar RNA, H/ACA box 8" |
| SNORD15A | "small nucleolar RNA, C/D box 15A" |
| SOWAHC | sosondowah ankyrin repeat domain family member C |
| SOX6 | SRY-box 6 |
| ST3GAL3 | "ST3 beta-galactoside alpha-2,3-sialyltransferase 3" |
| STAC3 | SH3 and cysteine rich domain 3 |
| STAP2 | signal transducing adaptor family member 2 |
| STARD8 | StAR related lipid transfer domain containing 8 |
| SULT1A1 | sulfotransferase family 1A member 1 |
| SYT11 | synaptotagmin 11 |
| TCF25 | transcription factor 25 |
| TEN1-CDK3 | TEN1-CDK3 readthrough (NMD candidate) |
| TEX30 | testis expressed 30 |
| TFDP1 | transcription factor Dp-1 |
| TGIF2-C20orf24 | TGIF2-C20orf24 readthrough |
| TMEM220-AS1 | TMEM220 antisense RNA 1 |
| TMEM242 | transmembrane protein 242 |
| TMEM86B | transmembrane protein 86B |
| TMEM91 | transmembrane protein 91 |
| TMEM98 | transmembrane protein 98 |
| TNNT1 | "troponin T1, slow skeletal type" |
| TPD52L1 | tumor protein D52 like 1 |
| TPI1P3 | triosephosphate isomerase 1 pseudogene 3 |
| TPM2 | tropomyosin 2 |
| TPP2 | tripeptidyl peptidase 2 |
| TSLP | thymic stromal lymphopoietin |
| TSPYL4 | TSPY like 4 |
| TTYH1 | tweety family member 1 |
| TUBB2B | tubulin beta 2B class IIb |
| UBAC2 | UBA domain containing 2 |
| UBAP1L | ubiquitin associated protein 1 like |
| UBE2E3 | ubiquitin conjugating enzyme E2 E3 |
| UBL3 | ubiquitin like 3 |
| UPF3A | "UPF3A, regulator of nonsense mediated mRNA decay" |
| YPEL3 | yippee like 3 |
| YTHDF3-AS1 | YTHDF3 antisense RNA 1 (head to head) |
| ZNF837 | zinc finger protein 837 |
